# Aging-Associated Differences in Epitranscriptomic m6A Regulation in Response to Acute Cardiac Ischemia/Reperfusion Injury in Female Mice

**DOI:** 10.3389/fphar.2021.654316

**Published:** 2021-08-03

**Authors:** Xuan Su, Yan Shen, Yue Jin, Il-man Kim, Neal L. Weintraub, Yaoliang Tang

**Affiliations:** ^1^ Vascular Biology Center, Medical College of Georgia, Augusta University, Augusta, GA, United States; ^2^ Anatomy, Cell Biology & Physiology, Indiana University School of Medicine, Indianapolis, IN, United States,

**Keywords:** aging, epitranscriptomics, M6A RNA methylation, myocardial ischemia/reperfusion, METTL3, FTO, pten

## Abstract

Elderly patients are more susceptible to ischemic injury. N6-methyladenosine (m6A) modification is the most abundant reversible epitranscriptomic modification in mammalian RNA and plays a vital role in many biological processes. However, it is unclear whether age difference impacts m6A RNA methylation in hearts and their response to acute myocardial ischemia/reperfusion (I/R) injury. In this study, we measured the global level of m6A RNA methylation as well as the expression of m6A RNA “writers” (methylation enzymes) and “erasers” (demethylation enzymes) in the hearts of young and elderly female mice undergone sham surgery or acute MI/R injury. We found that m6A RNA level and associate modifier gene expression was similar in intact young and old female hearts. However, young hearts show a significant reduction in m6A RNA while elderly hearts showed only a slight reduction in m6A RNA in response to acute I/R injury. To explore the mechanism of differential level of m6A RNA modification, we use qRT-PCR and Western blotting to compare the mRNA and protein expression of major m6A-related “writers” (Mettl3, Mettl14, and WTAP) and ‘erasers” (ALKBH5 and FTO). Mettl3 mRNA and protein expression were significantly reduced in both young and elderly hearts. However, the levels of FTO’s mRNA and protein were only significantly reduced in ischemic elderly hearts, and age-related downregulation of FTO may offset the effect of reduced Mettl3 on reduced m6A RNA level in the hearts of aging mice hearts with acute I/R injury, indicating aging-related differences in epitranscriptomic m6A regulation in hearts in response to acute I/R injury. To further investigate specific I/R related targets of Mettl3, we overexpressed Mettl3 in cardiomyocyte line (HL1) using lentiviral vector, and the m6A enrichment of Bcl2, Bax and PTEN were quantified with m6A RIP-qPCR, we found that m6A modification of PTEN mRNA decreased after *in vitro* hypoxia/reperfusion injury (iH/R) while Mettl3 augments m6A levels of both Bax and PTEN after iH/R, indicating that Bax and PTEN are target genes of Mettl3 under iH/R stress.

## Introduction

The aging process is associated with progressive functional decline and reduced stress tolerance (Leon and Gustafsson, 2016). Compared with a young adult, the elderly are at increased risk for various diseases, including neurodegeneration, diabetes, osteoporosis, cancer, and cardiovascular disease (Xu et al., 2016; Xu et al., 2017; Sun et al., 2019). Despite advances in pharmacologic and interventional therapies, cardiovascular disease remains the leading cause of death in the United States, with an increased prevalence and mortality during aging (Maggioni et al., 1993; Aguirre et al., 1994; Turner et al., 2013; Benjamin et al., 2018). One reason for the poor outcome in aged hearts might be reduced myocardial resistance to ischemia-reperfusion (I/R) injury (Li et al., 2015), which is a complex mechanism involving aberrant mitochondrial function (Korzick and Lancaster, 2013), Ca^2+^ sensitivity (Hano et al., 1995), oxidative stress response (Abete et al., 1999), and cardioprotective signaling (Randhawa et al., 2018).

N6-methyladenosine (m6A) modification is the most abundant reversible posttranscriptional modification on mammalian mRNA (Fu et al., 2014; Zeng et al., 2018), and it is dynamically regulated by a family methyltransferase enzymes (writers) that catalyze addition of m6A. Family members include Methyltransferase Like 3 (METTL3), Methyltransferase Like 14 (METTL14), WT1 Associated Protein (WTAP). M6A modifications are also regulated by demethylase enzymes (erasers) that catalyze removal of m6A; these enzymes include alpha-ketoglutarate-dependent dioxygenase AlkB homolog 5 (ALKBH5) and fat mass and obesity-associated protein (FTO) (Jia et al., 2019) (Roignant and Soller, 2017; Zhao et al., 2017; Tuncel and Kalkan, 2019). m6A RNA modifications have been reported to modulate stem cell proliferation and differentiation (Klungland et al., 2016), tumorigenesis (Cui et al., 2017), heat shock response (Zhou et al., 2015), and DNA damage response (Xiang et al., 2017). These findings indicate that m6A RNA methylation participates in numerous biological processes (Engel et al., 2018).

The role of m6A RNA modifications in cardiac aging has not been investigated. Since aged patients are more suspectable to myocardial ischemia-reperfusion (I/R) injury, we hypothesized that key functional responses controlled by m6A RNA methylation, such as heat shock and DNA damage responses, differ in aged compared to young hearts and contribute to the disparate responses. To begin to test this hypothesis, we compared age-related differences in m6A RNA methylation and the expression of m6A RNA modification enzymes under physiological and stress conditions to identify candidate genes responsible for the increased susceptibility to acute I/R injury in aged hearts. We detected significant aging-related differences in epitranscriptomic m6A regulation in acute I/R hearts which might contribute to the age-related intolence to myocardial ischemia. Further, to identify specific targets of Mettl3 during I/R, we overexpressed Mettl3 in cardiomyocyte line (HL1) using lentiviral vector and found that Bax and PTEN are target genes of Mettl3 under *in vitro* conditions of hypoxia/reperfusion injury.

## Methods

### Animals

The experimental protocols were conducted following the Guide for the Care and Use of Laboratory Animals (National Research Council Committee for the Update of the Guide for the, and A. Use of Laboratory Animals, 2011) and approved by the Institutional Animal Care and Use Committee of the Medical College of Georgia at Augusta University. Two to three month-old C57BL/6 female mice (The Jackson Laboratory) and 20 to 24 month-old C57BL/6 female mice (National Institute on Aging) were used in this study. To avoid the influence of gender, female mice were used in the present study. All mice were maintained under controlled environmental conditions and allowed to acclimate for at least 2 weeks in our facilities before use.

### Mouse Model of Acute Myocardial Ischemia and Reperfusion (I/R)

The surgical procedure used to induce myocardial I/R in mice was performed as previously described (Chen et al., 2019). After anesthetization with the intraperitoneal injection of 100 mg/kg body weight (BW) of ketamine and 10 mg/kg BW of xylazine, endotracheal intubation was performed with a 24-gauge catheter and mice were ventilated with room air at a rate of 195 breaths/min using a Harvard Rodent Ventilator (Model 55–7058, Holliston, MA, United Kingdom). A left thoracotomy was performed at the level of the fourth intercostal space, and the heart was briefly everted from the thoracic cavity by a pericardial incision. An 8–0 nylon suture was placed under the left coronary artery and then threaded through plastic PE10 tubing to form a snare for reversible left coronary artery occlusion. The thoracic cavity, pectoralis muscles, and skin were then closed in order using 6–0 nylon suture. Ischemia was induced by occlusion of the left coronary artery for 45 min, followed by removing the tubing to induce reperfusion for 2 hrs. Sham-operated control mice were subjected to the thoracotomy procedure without the ischemia and reperfusion (*n* = 5 per group). The chest was closed and the mice allowed to recover. Mice were sacrificed at 2 hours after reperfusion.

### Total RNA Extraction

Total RNA was extracted by RNAzol (Molecular Research Center, Inc., Cincinnati, OH, United States) from the homogenates of the peri-ischemic areas of the heart tissues following the manufacturer’s protocol. RNA quantity and purity were assessed with a Nanodrop 2000 spectrophotometer (Thermo Scientific).

### Quantification of m6A Modification

For quantification of m6A in total RNA, we used the EpiQuik m6A RNA Methylation Quantification Kit (Colorimetric, Epigentek) following the manufacture’s instructions. Briefly, 300 ng of RNA per sample was added to the assay well and incubated with a binding solution. Capture antibody, detection antibody, and enhancer solutions were then added to assay wells separately in a suitable diluted concentration as recommended by the manufacturer’s protocol. After that, the developer solution and stop solution were added. The m6A levels were quantified using the colorimetrical analysis by reading the absorbance at 450 nm, and then calculations were performed based on corresponding control samples.

### Reverse Transcription Reaction and Quantitative Real-Time PCR

Reverse transcription was performed with 1 μg total RNA using RevertAid First Strand cDNA Synthesis kits (Thermo Scientific). The cDNA was used to perform qRT-PCR on a CFX96 Touch Real-Time PCR Detection System (Bio-Rad Laboratories) using PowerUp SYBR Green Master Mix (Thermo Fisher). Amplification was performed at 50°C for 2 min, 95°C for 2 min, followed by 50 cycles of 95°C for 15 s, and 60°C for 1 min with the primers listed in Table 1. The relative gene expression levels were normalized to β-actin and subsequently calculated using the comparative cycle threshold method (ΔΔCt).

**TABLE 1:** Primer list.

Gene	Sequence (5′-3′)
β-actin FWD	AGA​GCA​TAG​CCC​TCG​TAG​AT
β-actin REV	GCT​GTG​CTG​TCC​CTG​TAT​G
Mettl3 FWD	GCT​TCG​CGA​GAG​ATT​GCA​G
Mettl3 REV	TAG​GCA​CGG​GAC​TAT​CAC​TAC
Mettl14 FWD	GGG​AAA​GAA​ACC​GAT​CCA​ATT​T
Metll14 REV	AGTAAAGCCGCCTCTGTG
WTAP FWD	CCC​TGA​AGA​AGA​AGG​AAA​GTC​A
WTAP REV	CTG​GCA​AAT​GGA​CCA​AGT​AAT​G
ALKBH5 FWD	AACAGCGCGGTCATCAA
ALKBH5 REV	CGA​GTC​GCT​GAA​GAA​AGA​CA
FTO FWD	GTC​AGA​GAG​AAG​GCC​AAT​GAA
FTO REV	CTC​TGC​TCT​TAA​GGT​CCA​CTT​C
mBcl2-3UTR m6A FWD	CTC​TAC​AGT​GGC​AAG​TGT​CTT​AG
mBcl2-3UTR m6A REV	AGGGTCTGCTACCCTCAG
mBax-3UTR m6A FWD	AAA​GAC​ACA​GTC​CAA​GGC​A
mBax-3UTR m6A REV	ACC​ATC​TTT​GTG​GCT​GGA​G
mPTEN-3UTR m6A FWD	GCC​ATC​AAA​TCC​AGA​GGC​TA
mPTEN-3UTR m6A REV	TGG​TGT​CAG​AAT​ATC​TAT​AAT​GAT​CAG​G

### Western Blot

The protein component of the heart tissue was lysed with RIPA lysis buffer (Alfa Aesar). After quantifying by BCA protein kit (Thermo Scientific), equal amounts of protein were loaded on a 10% or 12% sodium dodecyl sulfate-polyacrylamide gel and separated by electrophoresis. Then, the protein was transferred to a nitrocellulose membrane. After blocking with Odyssey blocking buffer (LI-COR Biosciences), membranes were incubated with rabbit anti-Mettl3 (1:1,000, Proteintech), rabbit anti-Mettl14 (1:1,000, ABclonal), rabbit anti-WTAP (1:1,000, Cell Signaling Technology), rabbit anti-ALKBH5 (1:1,000, NOVUS Biologicals), rabbit anti-FTO (1:1,000, NOVUS Biologicals), and mouse anti-GAPDH (1:5,000, MilliporeSigma) overnight on a rocker at 4°C. Membranes were then incubated for 1 h at room temperature with IRDye 680 goat anti-rabbit IgG and IRDye 800 Goat anti-mouse IgG (1:10,000, LI-COR Biosciences). The probed blots were scanned using an Odyssey infrared imager (LI-COR Biosciences).

### Cell Culture

Mouse HL1 cardiomyocytes (a generous gift from Dr. Claycomb) were cultured in Claycomb medium (Sigma-Aldrich) containing 10% fetal bovine serum (Thermo Fisher Scientific), 100 μg/ml penicillin/streptomycin (Thermo Fisher Scientific), 0.1 mM norepinephrine (Sigma-Aldrich) and 2 mM GlutaMAX (Thermo Fisher Scientific). Cells were plated in 6-well cell culture plates pre-coated with 0.5% (ml/ml) fibronectin and 0.2% gelatin (Sigma-Aldrich).

### Construction of Mettl3 Overexpression Lentiviral Vector for Cardiomyocyte Line Cell Transduction

To investigate specific targets of Mettl3 in cardiomyocyte cell lines following simulated I/R we constructed a pLV-EF1a-Flag-Mettl3-Neo plasmid by amplifying the full-length wild type human flag-Mettl3 cDNA from pcDNA3/Flag-METTL3 (a gift from Chuan He, Addgene plasmid # 53739) by PCR using the primers: forward 5′-GGT​GTC​GTG​AGG​ATC​GCC​ACC​ATG​GAT​TAC​AAG​GAT​GAC-3′, reverse 5′-CGC​CCT​CGA​GGA​ATT​CTA​TAA​ATT​CTT​AGG​TTT​AGA​GAT​GAT​ACC​ATC​TGG​GT-3’. The amplified DNA bands were then excised and purified from gel using QIAquick PCR gel purification kit (Qiagen). Purified DNA was in-framed cloned into the BamH1 and EcoR1 sites of pLV-EF1a-IRES-Neo (a gift from Tobias Meyer, Addgene plasmid # 85139) using Infusion Cloning kit (Clontech).

Lentiviral particles were packaged in HEK293FT cells (Thermo Fisher) by co-transfecting pLV-EF1a-Flag-Mettl3-IRES-Neo or empty pLV-EF1a-IRES-Neo plasmids and helper plasmids including pMD2. G (Addgene #12259) and psPAX2 (Addgene #12260) using lipofectamine 3000 reagents (Thermo Fisher). After 48 h, the viral supernatant was collected and filtered through a 0.45 μm filter to remove cells debris. The lentiviral vectors were purified by adding polyethylene glycol 6000 (PEG6000) (final concentration 8.5%) and NaCl (final concentration 0.4 M) to the supernatant as previously reported (Jin et al., 2020). When HL1 cells reached 80% confluence, they were infected with purified empty lentivirus and Mettl3 lentivirus in complete culture medium containing 8 μg/ml of polypropylene. Three days after lentiviral transduction, G418 (500 μg/ml) was added for cell selection to get HL1 ^ctrl^ (HL1 with empty lentivirus infection), and HL1 ^Mettl3^ (HL1 with Mettl3 lentivirus infection).

### *In vitro* Hypoxia/Reperfusion Model

To mimic the pathological process of ischemia/reperfusion *in vitro*, we developed a protocol in which HL1 cardiomyocytes were subjected to oxygen-glucose deprivation (0% O_2_ for 45 min in serum-free, glucose-free DMEM media using an anoxia pouch) followed by application of oxidative stress (500 µM H_2_O_2_ for 2 h).

### RNA Fragmentation and MeRIP-qRT-PCR

RNA was extracted from HL1 ^ctrl^ and HL1 ^Mettl3^ with/without iH/R treatment using RNAzol®RT (Molecular Research Center, Inc.). RNA was then fragmented using NEBNext® Magnesium RNA Fragmentation Module (New England Biolabs). The m6A methylRNA immunoprecipitation (MeRIP) was performed using EpiMark® N6-Methyladenosine Enrichment Kit according to instructions with modification as follows. Briefly, 2 µl N6-Methyladenosine Antibody was added to protein G magnetic beads (Thermo Fisher) and incubated at 4°C for 2 h. Following two washes in reaction buffer, the RNA was added to the antibody–bead mixture containing RNasin Plus RNase Inhibitor (Promega) and incubated at 4°C for 2 h. Next, RIP-enriched RNAs were isolated from the antibody-immobilized protein G beads using Monarch RNA cleanup binding buffer from Monarch RNA Cleanup kit (New England Biolabs). RT-qPCR was performed to evaluate the relative abundance of specific mRNA in m6A RIP complexes, as well as input samples from HL1 ^ctrl^ and HL1 ^Mettl3^ with/without iH/R treatment. We assessed the m6A level of Bcl2, Bax and PTEN, which are recognized as key regulators of cardiomyocyte apoptosis and survival (Shukla et al., 2011). We designed RT-qPCR primers according to m6A-Atlas (www.xjtlu.edu.cn/biologicalsciences/atlas), which presents a detailed analysis of the m6A epitranscriptome (Tang et al., 2021).

### Statistical Analysis

GraphPad Prism vision 8.0 software was used for the statistical analysis. Results were presented as the mean ± standard error of the mean (SEM). All results were analysed by one-way ANOVA with multiple comparisons, and Tukey's multiple comparison test was applied. The two-tailed Student’s *t*-test was performed to compare two groups. A *p*-value of less than 0.05 was considered statistically significant.

## Results

### Global m6A RNA Methylation in the Heart did Not Differ According to Age Under Physiological Conditions

**FIGURE 1:**
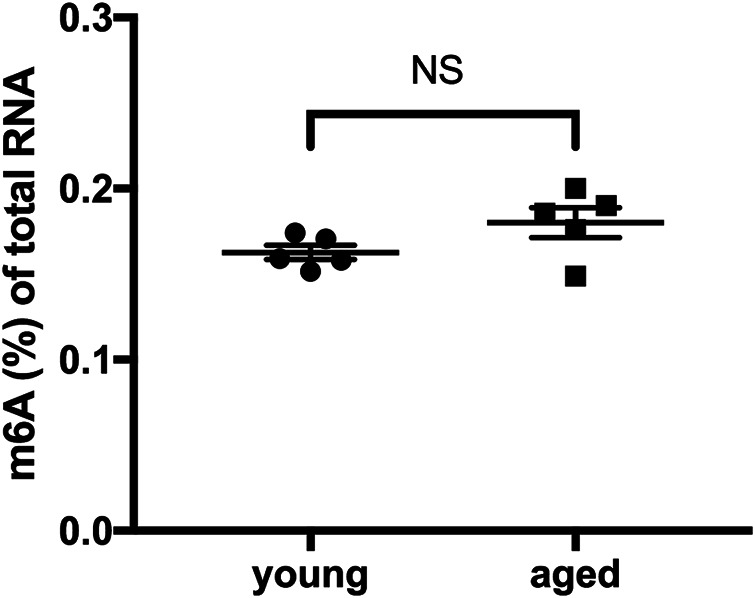
Global m6A RNA methylation in the heart did not differ according to age under physiological conditions. *n* = 5. Results are presented as mean ± standard error of the mean (SEM). NS, *p* > 0.05.

We quantified and compared m6A levels in total RNA isolated from young and aged mouse hearts under physiological conditions. No significant difference in levels of m6A RNA was observed between young hearts and aged hearts (Figure 1).

### Expression of m6A RNA Methylases and Demethylases in the Heart did Not Differ According to Age

**FIGURE 2:**
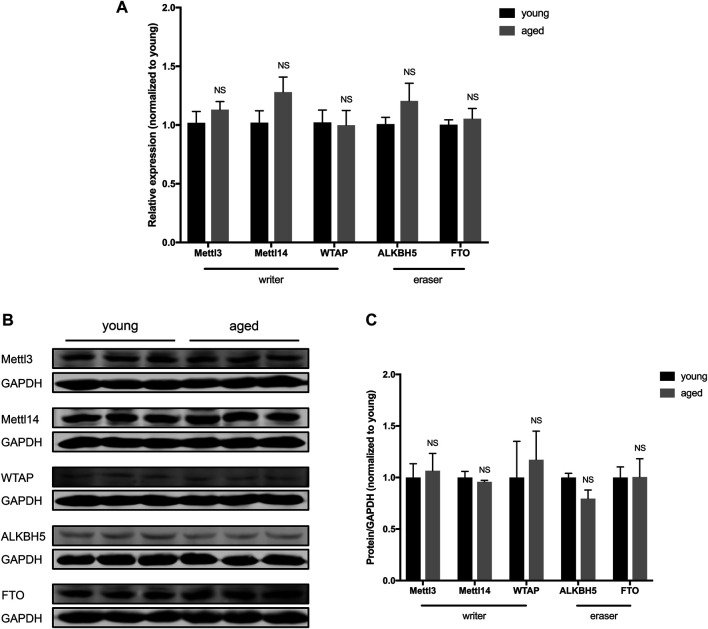
Expression of m6A RNA methylases and demethylases in the heart did not differ according to age. **(A).** Quantification of mRNA for m6A RNA methylases and demethylases, *n* = 5. **(B)**. Representative immunoblots for m6A RNA methylases and demethylases. **(C)**. Densitometry quantification of protein for m6A RNA methylases and demethylases, *n* = 3. Results are presented as mean ± standard error of the mean (SEM). NS, *p* > 0.05.

To investigate whether there is a difference in the expression of enzymes that control m6A RNA modifications, we examined the expression of “writers” (methyltransferases, including Mettl3, Mettl14 and WTAP) and “erasers” (demethylases, including Alkbh5 and FTO) in young and aged hearts. The expression of Mettl3, Mettl14, WTAP, Alkbh5 and FTO showed no significant difference at the mRNA level in aged hearts compared to young hearts (Figure 2A). At the protein level, there was also no significant difference in the expression of Mettl3, Mettl14, WTAP, Alkbh5 or FTO in young and aged hearts (Figures 2B,C). Collectively, these data suggest the expression of m6A RNA methylases and demethylases was stable in hearts during aging under physiological conditions.

### Age-Related Differences in Global m6A RNA Methylation in Response to Acute Myocardial I/R Injury

**FIGURE 3:**
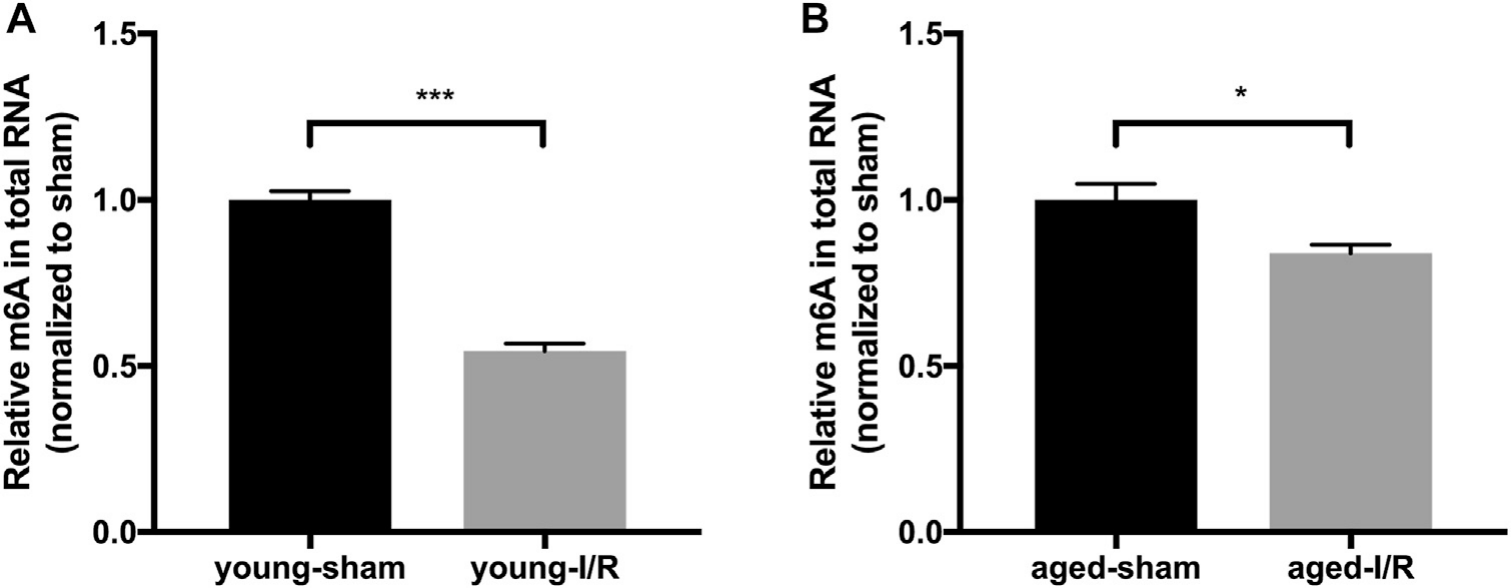
Age-related differences in global m6A RNA methylation in response to acute myocardial I/R injury. *n* = 5. Results are presented as mean ± standard error of the mean (SEM). *p < 0.05; ***p < 0.001.

To test the impact of aging on m6A RNA modifications in response to acute myocardial I/R injury, we examined the m6A levels in RNA extracted from young and aged hearts at 2 h after I/R injury. In young mice, I/R injury led to a 45% decrease of m6A RNA levels in comparison with sham control, while there was only a 16% decrease detected in aged mice under these conditions (Figures 3A,B). These results suggest that acute myocardial I/R injury-induced decrease of m6A RNA level occurs in both young and aged mice. Notably, however, the magnitude of reduction of m6A RNA is less in aged hearts following acute myocardial I/R injury.

### Age-Related Differences in the Expression of m6A RNA Methylases and Demethylases in Response to Acute Myocardial I/R Injury

**FIGURE 4:**
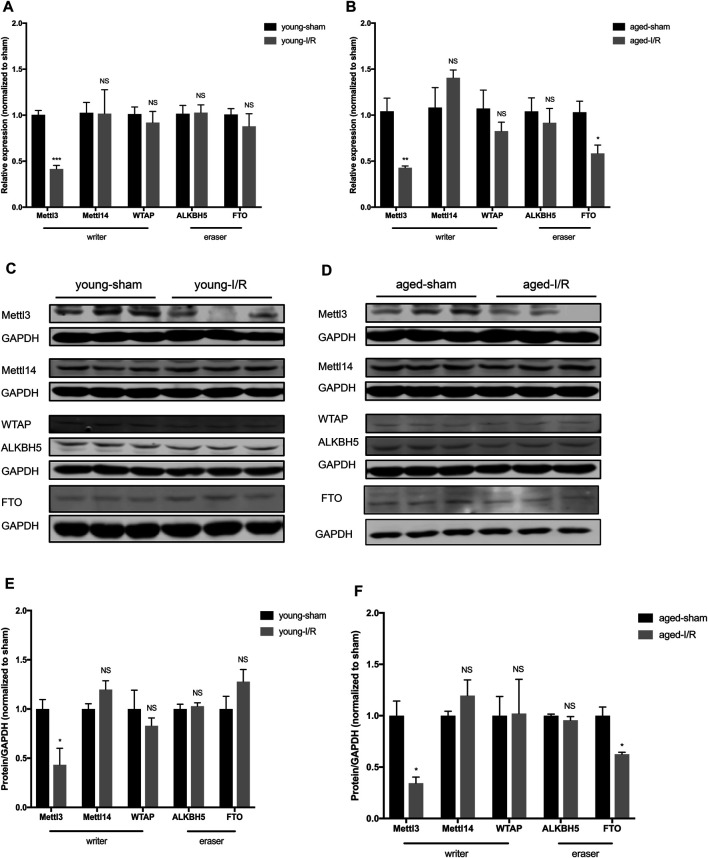
Age-related differences in the expression of m6A RNA methylases and demethylases in response to acute myocardial I/R injury. **(A)**. Quantification of mRNA level of m6A RNA methylases and demethylases in young mice, n = 4 to 5. **(B)**. Quantification of mRNA for m6A RNA methylases and demethylases in aged mice, *n* = 4 to 5. **(C)**. Representative immunoblots for m6A RNA methylases and demethylases in young mice undergoing sham and I/R injury. **(D)**. Representative immunoblots for m6A methylases and demethylases in aged mice undergoing sham and I/R injury. **(E)**. Densitometry quantification of protein for m6A RNA methylases and demethylases in young mice, *n* = 3. **(F)**. Densitometry quantification of protein for m6A methylases and demethylases in aged mice, *n* = 3. Results are presented as mean ± standard error of the mean (SEM). NS, *p* > 0.05; *p < 0.05; **p < 0.01; ***p < 0.001.

To identify the expression pattern of m6A RNA methylases and demethylases in governing the age-related differences in m6A RNA methylation post-acute myocardial I/R injury, we measured the expression of three m6A RNA methylases (Mettl3, Mettl14, and WTAP) and two demethylases (Alkbh5 and FTO). The expression of Mettl14, WTAP and Alkbh5 (mRNA or protein) did not change significantly in young versus aged hearts in response to acute cardiac I/R injury. After acute cardiac I/R injury, both young and aged hearts exhibited reduced levels of Mettl3 mRNA and protein by qRT-PCR and Western blotting (Figure 4A–F), in accordance with reduced m6A RNA levels (Figures 3A,B). In addition, we only observed significant downregulation of FTO mRNA and protein levels in aged ischemic hearts (Figures 4B,D,F), suggesting that age-related downregulation of FTO may offset the effect of reduced Mettl3 on m6A RNA levels following acute I/R injury.

### *In vitro* Hypoxia/Reperfusion Stress Regulates m6A Levels of Key Anti-apoptotic and Pro-apoptotic Genes

**FIGURE 5:**
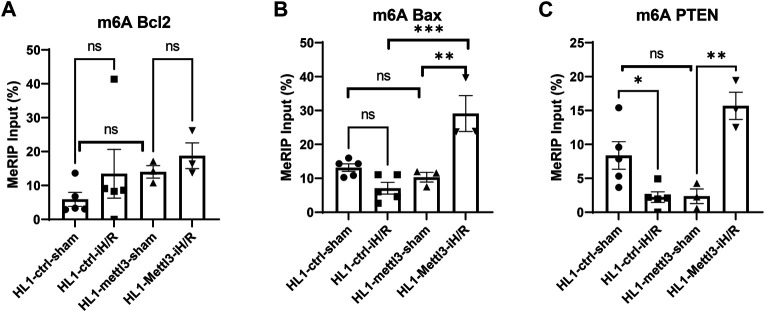
*In vitro* hypoxia/reperfusion (iH/R) stress regulates m6A levels of key anti-apoptotic and pro-apoptotic genes. (**A–C**). Ctrl and Mettl3 mofified HL1 cardiomyocytes were subjected to sham or iH/R, and total RNA was subjected to m6A RIP, followed by RT-qPCR using primers for the indicated pro-apoptotic genes (Bax and PTEN) and anti-apoptitic gene (Bcl2). Values are normalized to input. *n* = 3–5, *p < 0.05; **p < 0.01; ***p < 0.001.

We selected Bcl2, Bax and PTEN to investigate the effects of iH/R on m6A methylation of key anti-apoptotic and pro-apoptotic genes, as public m6A databases show that they have m6A peaks near the stop codon or in their 3′UTR region (Chen et al., 2020; Yang et al., 2020). As shown in Figures 5A–C, iH/R stress significantly reduced m6A methylation of PTEN compared to sham-treated cardiomyocytes while having smaller and less consistent effects on the m6A level of Bax and Bcl2 mRNAs.

Next, we tested the effects of Mettl3 overexpression on regulating m6A methylation levels of the three key anti-apoptotic and pro-apoptotic genes in the absence and presence of iH/R. As shown in Figures 5A–C, HL1 ^Mettl3^ did not exhibit differences in m6A levels of Bcl2, Bax, or PTEN levels compared to control HL1 ^ctrl^ in sham-treated cells (i.e., in the absence of iH/R). However, iH/R led to significantly increased m6A levels of both Bax and PTEN mRNA in HL1 ^Mettl3^, indicating that Mettl3-mediated m6A methylation is selectively dependent on hypoxic stress.

## Discussion

In the present study, we studied the age-related differences in m6A RNA methylation in the hearts of young and aged female mice under physiological and pathological conditions. Our results suggest that aging affects global m6A RNA methylation, and associated methylation and demethylation enzyme expression, only under pathological conditions. Aging-associated FTO downregulation might be associated with impaired myocardial ischemia tolerance in elderly patients.

We employed a model of acute myocardial I/R injury in young and aged mice. The age of sexual maturity in C57BL/6 mice is around 35 days. Thus, the young age group (2–4 months of age) could be considered as developed young adults. According to the life-span, C57BL/6 mice 18–24 months of age can be considered aged mice, while mice older than 24 months of age are senescent. A previous study demonstrated that age-related ischemic intolerance develops well before senescence, and there is a modest improvement of ischemic tolerance in senescent mice (Willems et al., 2005). Thus, mice aged between 20 and 24 months were chosen in our study to investigate the effect of aging on ischemic tolerance and m6A levels in comparison to young mice.

As the most prevalent RNA modification, m6A marks can dynamically regulate RNA metabolism and cardiomyocyte responses to stress, such as ischemia/reperfusion.

Our results demonstrate that the levels of global m6A RNA decreased after myocardial I/R injury in both young and aged mice, in conjunction with the downregulation of Mettl3, a key RNA methyltransferase, suggesting that Mettl3 plays a role in response to the myocardial I/R injury. Supporting our findings, decreased levels of m6A and Mettl3 have been reported previously in acute stress models in mouse brain (Engel et al., 2018) and kidney (Wang et al., 2019). Additionally, Mettl3 was reported to modulate cardiac homeostasis through m6A modifications, and Mettl3 knockdown in cardiomyocytes promoted the progression of heart failure (Dorn et al., 2019). On the other hand, METTL3 was reported to be upregulated in neonatal cardiomyocytes in subjected to hypoxia/reoxygenation (Song et al., 2019). As for the difference of Mettl3 expression in hearts between the present and prior study, we assume that this can be explained on the basis of differences between neonatal versus adult hearts, which are well documented with regard to cardioprotective responses (Chen et al., 2017; Ionio et al., 2021).

Previous studies reported that aged mice are less tolerant of acute ischemic stress than young mice (Headrick, 1998; Willems et al., 2005). It is interesting to note that a significant decrease of FTO was only observed in the aged mouse hearts subjected to I/R injury, in conjunction with less decrease of m6A in total RNA in comparison to young mice. A recent study reported that FTO is downregulated in failing human and mouse hearts, and FTO plays a protective role under ischemia by selectively demethylating cardiac contractile transcripts (Mathiyalagan et al., 2019). Moreover, Zhang et al. (2019) reported that FTO is required for the maintenance of bone mass, and loss of FTO leads to increased susceptibility to apoptosis in murine osteoblasts. Therefore, age-related loss of FTO might be associated with ischemia intolerance in aged hearts as well as other aspects of aging-related organ and tissue dysfunction.

To investigate dynamic changes in m6A modifications in a cardiomyocyte cell line under iH/R, we analyzed m6A-modifications of key anti-apoptotic (Bcl2) and pro-apoptotic (Bax and PTEN) genes using m6A RIP-RT-qPCR. We analyzed the m6A methylation level of these gene transcripts by designing PCR primers targeting unique m6A sites identified from m6A-Atlas (www.xjtlu.edu.cn/biologicalsciences/atlas), which features a high-confidence collection of 442,162 reliable m6A sites identified from seven base-resolution technologies and the quantitative (rather than binary) epitranscriptome profiles estimated from 1,363 high-throughput sequencing samples (Tang et al., 2021). We found that iH/R injury significantly reduced the m6A methylation level of PTEN transcript, without significantly changing m6A methylation of Bcl2 or Bax. In addition, to investigate specific I/R related targets of Mettl3, we employed a lentiviral vector to overexpress Mettl3 in HL1 cells. We found that Mettl3 overexpression does not significantly change m6A Bcl2, m6A Bax or m6A PTEN level in comparison to control vector modified cardiomyocyte lines under baseline conditions. However, Mettl3 overexpression significantly increased both m6A PTEN and m6A Bax levels after iH/R injury, without consistently impacting m6A Bcl2 levels, suggesting that the effect of Mettl3-medated m6A methylation modulation is selective and iH/R dependent. A recent study demonstrated that METTL3 knockdown strongly induces the mitochondrial apoptotic pathways in lung cancer cells by increasing the levels of cleaved caspase3 and PARP (Wei et al., 2019), and Luo et al. (2021) recent reported that METTL3 knockdown induced cell apoptosis and senescence of dental pulp stem cells. M6A modification has been reported to facilitate mRNA degradation via binding to a specific degradative protein complex (Yue et al., 2015). Thus, Mettl3 overexpression-induced increases in m6A of Bax and PTEN mRNAs might protect cardiomyocytes from iH/R induced apoptosis via downregulation of pro-apoptotic gene expression. We plan to study this possibility in our future experiments.

There are several limitations of this study. First, we measured m6A levels in total RNA instead of purified mRNA. However, most of the m6A measured in total RNA is in rRNA, which is generated by different enzymes. For example, m6A in structural RNAs and U6 snRNAs can be methylated by METTL16, and METTL5 mediates m6A modification of 18 S rRNA and participates with ZCCHC4 in 28S rRNA modifications (Pan et al., 2021). We attempted to measure m6A in mRNA in heart tissue but could not isolate enough purified mRNA after two rounds of polyA selection for m6A RNA quantification using an ELISA-based protocol. Second, we did not perform m6A RNA-seq, which could provide more comprehensive information. To mitigate this weakness, we investigated specific targets of Mettl3 using m6A RIP-qPCR, and demonstrated that Bax and PTEN are target genes of Mettl3 under iH/R stress. Finally, we only investigated female mice in this study, and estrogen has significant effects on cardiac ischemia. Thus, it is possible that different m6A RNA profiles might be observed in male mice in the setting of I/R.

In conclusion, for the first time we demonstrate age-related differences in m6A RNA methylation in response to acute myocardial I/R injury. In addition, age-related downregulation of FTO expression in response to acute myocardial I/R injury was only detected in aged hearts. Most importantly, we identified Bax and PTEN as target genes of Mettl3 under iH/R stress. Further research is necessary to understand the mechanisms of FTO dysregulation in ischemic aging hearts, and the relationship between FTO and cardiac apoptosis post cardiac injury.

## Data Availability

The original contribution presented in the study are included in the article/supplementary material, further inquiries can be directed to the corresponding author.
